# Factors associated with return to work among disability claimants with depression

**DOI:** 10.4102/ajod.v14i0.1737

**Published:** 2025-09-22

**Authors:** Anisha Maharaj, Munira Hoosain, Carl Lombard, Lana van Niekerk

**Affiliations:** 1 Department of Occupational Therapy, Faculty of Medicine and Health Sciences, Stellenbosch University, Cape Town, South Africa; 2 Department of Epidemiology and Biostatistics, Faculty of Medicine and Health Sciences, Stellenbosch University, Cape Town, South Africa

**Keywords:** case management, insurance, mental health, occupational therapy, vocational rehabilitation

## Abstract

**Background:**

Depression is a significant contributor to the burden of disease globally and is often associated with reduced work productivity and permanent disability.

**Objectives:**

This study aimed to identify factors associated with return to work among disability claimants with depression at one insurer in South Africa.

**Method:**

A retrospective cohort study was conducted with 514 disability claims. Univariate tests for association (Chi-square and Fisher) were performed to test the significance of the association between the primary outcome (disability benefit terminated as a result of return to work) and demographic and workplace factors.

**Results:**

The majority of disability claimants were women (71.6%). Women and claimants under the age of 60 years at the time of benefit termination had a high probability of returning to work, 80% and 99% respectively. Of the 39% of claimants who had a high level of workplace support during the disability process, 95% also returned to work. Nearly half (48%) of the disability claimants returned to work in less than 6 months, while most of those with a duration of disability longer than 24 months did not return to work (79%).

**Conclusion:**

Female gender, lower age (under 60 years), high workplace support and shorter duration of disability were all positively associated with return to work for disability claimants with depression.

**Contribution:**

This study provides insights into factors associated with successful return to work for disability claimants with depression, which can guide case management and rehabilitation for this population.

## Introduction

Psychiatric conditions are a significant contributor to the burden of disease in all regions of the world (Tomlinson et al. 2009). Depression has been identified as one of the most concerning health problems, as it is often associated with reduced productivity at work and permanent disability (Ramano, Buys & De Beer 2016; Tomlinson et al. 2009). Depression results in work absenteeism and loss of productivity while the employee is present at work (Ramano et al. 2016). It has also been found that individuals with depression have higher unemployment rates, increased workplace absences, decreased job retention and overall productivity impairments at work (Beck et al. 2014; De Vries et al. 2018; Lerner & Henke 2008), which may lead to a disability claim. There is global unanimity regarding the rising prevalence of depression in the workplace and the rise in mental health disability claims (Alexander Forbes 2019; Ervasti et al. 2017; Mokoka, Rataemane & Dos Santos 2012; Momentum Corporate 2019; Wisenthal et al. 2019).

The World Health Organization (WHO) estimates that depression will be the leading burden of disease by 2030, with a current prevalence of between 10% and 15% in the general population (Ramano et al. 2016; WHO 2011). A systematic review of cohort studies (*n* = 11) on the association between predictive factors and return to work among employees with depression by Ervasti et. al. identified seven domains of predictive factors in the Netherlands, Canada, Denmark, Sweden and Japan (Ervasti et al. 2017). These factors included sociodemographic, workplace, health behaviour, clinical and illness, psychosocial, personality and labour market factors. Their study is the first systematic review to provide quantitative estimates of factors contributing to return to work following depression. Their definition of return to work was based on the end of disability benefits. The conclusion from the review was that ‘higher age, Psychiatric and somatic comorbidity, more severe depression, and low conscientiousness were predictive of slower return to work after depression’ (Ervasti et al. 2017:36). Clinical and illness-related factors were the most consistent predictors of return to work following depression. Clinical and illness-related factors included the treatment of depression; within an occupational therapy framework, this includes vocational rehabilitation with the goal of return to work.

Vocational rehabilitation interventions include cognitive work hardening (CWH) and the Progressive Goal Attainment Programme (PGAP). Cognitive work hardening is an evidence-based treatment modality based on the principles of physical work hardening and applied to the domains of mental health to restore cognitive functionality and work abilities required for successful return to work. This is usually carried out by an occupational therapist through graded activities that simulate the disability claimant with depression (DCWD)’s job tasks and demands (Wisenthal et al. 2018), while PGAP aims to improve perceptions and cognition and improve work performance (De Wit et al. 2022).

Disability claimant with depression often have longer durations of disability benefit payments and require more in-depth and regular reviews with a reduced rate of return to work when compared to disability claimants with physical conditions (Adler et al. 2006; Alexander Forbes 2019). This may be related to the multidimensional and persistent impact of depression on functioning (Adler et al. 2006). Mental health in the workplace, more specifically depression, has been identified as a public health priority and a recommended focus of research (Søvold et al. 2021; Wisenthal & Krupa 2013; WHO 2022). There is limited evidence about factors associated with return to work in low- and middle-income countries (LMICs) (Mullerpatan & Jadhav 2025). This is particularly relevant for DCWD, as they may have difficulty accessing mental health services because of the limited number of mental health service providers in LMICs, language and cultural barriers, as well as social stigma and economic hardships (Hoosain, Mayet-Hoosain & Plastow 2023; Jansen Van Vuuren, Okyere & Aldersey 2021; WHO 2017). It is therefore important to identify the factors associated with return to work for DCWD in these settings.

By identifying the factors associated with successful return to work for DCWD, disability claims assessors can identify claimants who are at a higher risk of not returning to work as a result of the disability and assist with early interventions and recommendations to the employee, employer and mental health service providers. If the factors associated with successful return to work among DCWD in LMICs are discussed and contextualised, it can inform future research and policies within the insurance industry and health sectors. It will assist in case management and motivation for further funding for vocational rehabilitation interventions and engagements with employers when DCWD return to work.

Therefore, the aim of this retrospective cohort study was to identify factors associated with return to work among DCWD at one insurer in South Africa.

## Research methods and design

### The research setting

In the context of this study, a disability claimant refers to an employee of an employer that offers insurance for medical conditions in the form of a disability benefit. A disability claim for the employee has been admitted, and a benefit is being paid in view of their inability to perform an occupation because of depression. Termination of a disability benefit was taken as an indicator of successful return to work.

The first author is an occupational therapist employed to do disability claim assessments for an insurance company in South Africa, providing income protection cover. This income protection cover, paid for by the employer, is often the only financial cover employees (and their families) have (Alexander Forbes 2019). A monthly disability benefit equivalent to 75% of their salary is paid to an employee, herein referred to as a DCWD, whereby, in the reasonable opinion of the insurer, ‘the illness i.e. depression has rendered the employee incapable of engaging for remuneration or profit in the occupation that he or she was performing immediately prior to his or her date of disablement or in any other occupation in the open labour market that he or she is (or could become) qualified for or suited to, taking into account his or her degree of disability, knowledge, training, education, ability and experience’ (Momentum Corporate 2019). The company insures claimants from South Africa as well as Namibia, Lesotho, Botswana, Mozambique, Eswatini and Zimbabwe.

### Study design

A retrospective cohort study was carried out to correlate exposure to factors associated with successful return to work for DCWD while also obtaining a preliminary measure of association between the identified factors (*Prospective, Retrospective, Case-control, Cohort Studies* – Stats Direct n.d.). A retrospective study design was deemed the most applicable because the first author had access to data to identify the factors associated with return to work of the full population who had been exposed (or not exposed) to the studied outcome of successful return to work. These data had been anonymised. Informed consent could thus not be obtained, but an ethics waiver was received from the Health Research Ethics Committee at Stellenbosch University.

### Study population and sampling

The disability claimants on the insurer’s database used in this study span all socioeconomic categories and economic sectors (Alexander Forbes 2019; Momentum Corporate 2019). Total population sampling was used. The study population comprised disability claimants with the primary claim cause of depression on the insurer’s group disability database for which a final claim decision had been made between January 2017 and December 2020. The period census yielded 517 DCWD. No sample size calculation was carried out prior to data collection, as the total study population was used as the sample. DCWD who had their disability terminated because of death were excluded (*n* = 3) as this did not address the study aim.

### Data collection

The factors to be analysed were selected based on existing evidence from high-income countries (Ervasti et al. 2017), as factors shown to be associated with the claim outcome of interest. A data capture sheet was developed to capture the factors identified from the insurer’s disability database.

The data were collected by the first author and a research assistant, who were both employed by the insurer as disability claims assessors and had access to the database. The data capture process was trialled and compared between capturers to evaluate the coverage of the data capture sheet and enhance inter-rater reliability. Following the successful completion of the trial, data collection continued for all claims.

### Variables

#### Primary outcome

The primary outcome was the disability benefit termination for return to work. At the point in time when the disability benefit is terminated, the disability file will be closed, and the disability claimant would need to have successfully returned to work for a minimum of 3 months with no recurrent disability (Momentum Corporate 2019). Return to work could be in the format of onsite work, working from home (WFH) or a hybrid format.

#### Factors

**Gender:** Categorised as men and women as self-proclaimed on the disability claimant’s signed declaration form, as this is how it was recorded in the claims dataset. No provision was made for gender non-binary in the dataset.

**Age:** Broken down into age brackets of 18–29 years, 30–39 years, 40–49 years, 50–59 years, and above 60 years of age on the date of disability benefit termination.

**Duration of disability:** Number of months from the DCWD’s date of disability to the disability benefit termination date for return to work or censored at death or at the end of follow-up (24 months).

**Level of education:** The highest level of education obtained by the DCWD.

**Strength rating:** The Dictionary of Occupational Titles (DOT) is commonly used in functional capacity evaluations (FCE), disability claims assessments and vocational rehabilitation (Opsteegh et al. 2010). The DOT classifies jobs into categories based on the physical demands of an occupation: *sedentary* (exerting up to 4.5 kg of force occasionally and/or a negligible amount of force frequently to handle objects; sedentary work involves mostly sitting, possibly with some walking or standing for short periods); *light* (exerting up to 9.5 kg of force occasionally and/or up to 4.5 kg of force frequently and/or a negligible amount of force constantly to handle objects); *medium* (exerting 9.6 kg – 22.7 kg of force occasionally and/or 4.5 kg – 11.3 kg of force frequently and/or greater than negligible up to 4.5 kg of force constantly to move objects) and *heavy* (exerting 22.7 kg or more of force occasionally and/or 11.3 kg of more of force frequently and/or 4.5 kg or more of force constantly to handle objects) (Opsteegh et al. 2010).

**Income disability bracket:** Disability benefit amount received by the DCWD. Broken down into income brackets of ZAR1000.00 - ZAR19 999.00, ZAR20 000.00 - ZAR39 999.00, ZAR40 000.00 - ZAR59 999.00 and above ZAR60 000.00. For comparison, $1.00 = ZAR18.00 / EUR1.00 = ZAR20.00 at the time of publication.

**Functional capacity evaluation:** A comprehensive set of standardised assessments performed to determine a claimant’s physical ability in relation to the elected occupation (Wind et al. 2006).

**Job demands:** Refers to the level of cognitive demands, namely higher level of cognitive demands, moderate level of cognitive demands and lower level of cognitive demands.

**Workplace support:** Refers to how supportive the employer is during the disability process and how willing the employer is to engage in the return-to-work process for the DCWD. This is categorised into high, moderate and low levels of support by the insurer.

**Vocational rehabilitation and/or case management:** Case management and vocational rehabilitation are used interchangeably in this study and indicate that the DCWD has undergone rehabilitation interventions with the aim of return to work while in receipt of a disability benefit.

**Type of vocational rehabilitation and/or case management:** Occupational therapy interventions include cognitive work hardening, a concept of applying work hardening concepts to the domains of mental health to develop cognitive skills required for work performance (Wisenthal et al. 2018), and the Progressive Goal Attainment Programme, which aims to improve perceptions and cognition and improve work performance (De Wit et al. 2022) as part of vocational rehabilitation. Psychological intervention included psychotherapy. Intervention can include one or more therapies at a time, usually recommended by the disability claims assessor and paid for by the insurer.

**Duration of case management:** Number of months from commencement of case management to termination of case management

### Data analysis

Descriptive statistics consisted of frequencies and percentages. The primary outcome, disability benefit termination for return to work, was cross-tabulated with various demographic and workplace factors. To test for a difference in the percentage of benefit terminations between the levels of a factor, the Chi-square test or Fischer exact test was used. The latter was used when the data were sparse for certain levels (*n* < 5). A significance level of 0.05 was used.

The time to termination of disability was analysed using a Kaplan–Meier failure function. This function estimates the cumulative benefit termination over the time of the study. Apart from the Kaplan–Meier curve with 95% confidence intervals, the cumulative terminations are reported for 6, 12, 18, 24 and 25 months as well as the study population at risk.

### Ethical considerations

The Health Research Ethics Committee (HREC) at Stellenbosch University approved this study (Ref: S21/08/155) on 17 December 2021.

A waiver of consent was provided by HREC as the degree of risk that this study posed to participants to whom the data were linked, as well as the degree of risk posed to participants in the waiving of consent, was no more than minimal; this study made use of retrospective (secondary) data, which was anonymised from initial data collection. In addition, it was impracticable to obtain consent because of the quantity of data. Lastly, the data in this study were aggregated and anonymised in the reporting of findings. Thus, no individual cases were reported on, and anonymity was upheld.

## Results

A total of 517 claims formed the sample for this study. Disability claimants for whom benefits were terminated as a result of death (*n* = 3) were excluded. Of the 514 claims analysed, 77.2% (*n* = 397) were terminated as the DCWD returned to work.

### Return to work

There were 397 participants (77.2%) who returned to work within the study follow-up of 24 months.

**FIGURE 1:**
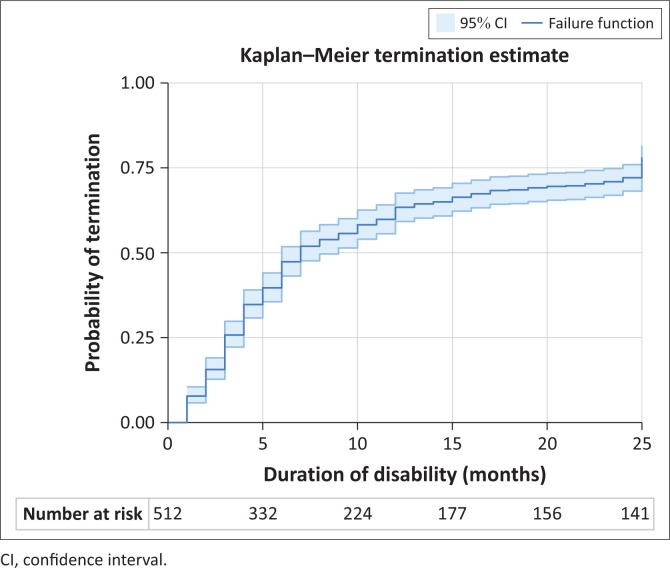
Termination of disability curve.

There were 3 deaths that censored the duration. In Figure 1, the termination of disability curve is shown, and in Table 1, the estimated termination probabilities at 6-month intervals are presented.

**TABLE 1:** Probability of termination of disability benefits over the study period.

Month	At risk	Prob. of termination	95% conf. int.	
			Lower	Upper
6	306	0.47	0.43	0.52
12	203	0.63	0.59	0.68
18	160	0.69	0.64	0.73
24	147	0.72	0.68	0.76
25	141	0.78	0.74	0.82

Prob., probability; conf. int., confidence interval.

The median time to termination was 7 months, and the rate of termination slowed down after 12 months on disability.

### Demographics of disability claimants with depression

Table 2 shows the prevalence of return to work for the demographic variables of the claimants.

**TABLE 2:** Demographics of disability claimants with depression.

Demographic factor	Group 1: DCWD who returned to work		Group 2: DCWD who did not return to work		*p*-value
	Number (*n* = 397)	%	Number (*n* = 117)	%	
**Gender**	-	-	-	-	0.023
Men	103	70.6	43	29.4	-
Women	294	79.9	74	20.1	-
**Age (years)**	-	-	-	-	< 0.001
18–29	41	100.0	0	0.0	-
30–39	116	99.2	1	0.8	-
40–49	135	98.5	2	1.5	-
50–59	82	98.8	1	1.2	-
Above 60	23	21.5	113	78.5	-
**Highest level of education**	-	-	-	-	0.008
Secondary school	18	58.1	13	41.9	-
High school qualification (Grade 12)	127	76.5	39	23.5	-
Diploma or Occupational Certificate	137	74.5	47	25.5	-
Bachelor’s degree or higher	78	85.7	13	14.3	-
Unknown	37	88.1	5	11.9	-
**Income disability benefit bracket (per month)**	-	-	-	-	0.035
ZAR1000.00-ZAR19 999.00	232	81.7	52	18.3	-
ZAR20 000.00-ZAR39 999.00	105	69.5	46	30.5	-
ZAR40 000.00-ZAR59 999.00	31	73.8	11	26.2	-
Above ZAR60 000.00	29	78.4	8	21.6	-
**Physical strength rating of job or occupation**	-	-	-	-	0.197
Sedentary	187	73.6	67	26.4	-
Light	125	79.6	32	20.4	-
Medium	67	79.8	17	20.2	-
Heavy	17	94.4	1	5.6	-
Unknown	1	100.0	0	0.0	-
**Job demands**	-	-	-	-	0.952
Low	14	77.8	4	22.2	-
Moderate	214	76.7	65	23.3	-
High	169	77.9	48	22.1	-

DCWD, disability claimant with depression.

Gender, age, level of education and disability benefit were significantly associated with successful return to work. From Table 2, we have male gender, age above 60 years, secondary education and receiving higher benefits as the demographic profiles associated with a lower probability of returning to work.

The physical strength rating of the job and the job demands were not significantly associated with successful return to work.

### Factors that predicted return to work or not among disability claimants with depression

In Group 1, with the outcome of DCWD who returned to work, most DCWD were between the ages of 18 years and 59 years (*n* = 374), but some were over 60 years old (*n* = 23). In Group 2, with the outcome of DCWD who had not returned to work, the majority of DCWD were over the age of 60 years (*n* = 113), with a small number of DCWD younger than 60 years (*n* = 4).

Of the 397 DCWD who returned to work in Group 1 outcome, 74.06% were women (*n* = 294), while only 25.94% were men (*n* = 103). Of the 117 DCWD who did not return to work in Group 2 outcome, 63.26% were women (*n* = 74), while 36.75% were men (*n* = 43). The number of women was higher in both outcome groups.

Disability claimants with depression who had returned to work (Group 1) had a higher level of workplace support (77.24%) when compared with DCWD who had not returned to work (Group 2), who had lower workplace support (22.76%) during the disability benefit process.

This study showed that age, gender, workplace support and duration of disability were all factors that influenced whether a DCWD would return to work among the two groups in terms of the outcomes.

### Factors associated with successful return to work for disability claimants with depression

Cross-tabulation of factors was conducted to identify the factors associated with benefit termination as a result of return to work. The Pearson Chi^2^ and probability ratio for each factor analysed are indicated in Table 3.

**TABLE 3:** Pearson Chi^2^ and probability ratio for each factor analysed for disability benefit termination.

Factor	Pearson Chi^2^ result	*p*-value
Workplace support	81.19	< 0.001
Functional capacity evaluation on file	65.05	< 0.001
Vocational rehabilitation and case management	0.44	0.506
Type of vocational rehabilitation and case management	1.01	0.799
Duration of vocational rehabilitation and case management	3.22	0.780

Workplace support and FCE on file were significant factors associated with the probability of returning to work. Specifically, a low level of work support and FCE on file are the work-related profiles that had a lower probability of returning to work. Workplaces that offer less support to their employees showed a lower termination for return to work (*p* < 0.001). Workplaces with low support had a termination rate of 34.21% (*n* = 13), compared to highly supportive workplaces with a successful return to work of 94.53% (*n* = 190).

Disability claimant with depression who had undergone an FCE had a lower disability benefit termination for return to work of 59.43% (*n* = 126) (*p* < 0.001). This could be an indication that DCWDs with more serious illnesses were more likely to require an FCE in the disability claim process. This, however, does show that FCEs did not support return to work.

Disability claimant with depression who had undergone vocational rehabilitation and/or case management had a successful return to work rate of 80% (*n* = 68). Of the DCWD who received vocational rehabilitation and/or case management, 81.25% (*n* = 52) received occupational therapy intervention, which included CWH and/or PGAP, while 75% (*n* = 15) of these DCWD received a combination of occupational therapy and psychotherapy interventions. DCWD who were part of a vocational rehabilitation programme for 4–6 months had a 100% rate of return to work (*n* = 26), while DCWD with 3 months or less had a return-to-work rate of 86.21%. disability claimants with depression who underwent 7–12 months of vocational rehabilitation and case management had a return-to-work rate of 68.75% (*n* = 11). Lastly, DCWD who underwent more than 12 months of vocational rehabilitation and/or case management had a return to-work-rate of 80% (*n* = 6). Women DCWD had a higher rate of engagement in case management (17.66%) when compared to men (13.70%).

## Discussion

This study expands on the sociodemographic, workplace, vocational rehabilitation and illness-related factors associated with successful return to work among DCWD at one insurer in South Africa. Gender (female), age (younger than 60 years) and high workplace support are all positive prognostic factors associated with return to work for DCWD.

The results confirm that women had a higher rate of return to work, as almost 80% of women returned to work following a termination of a disability claim for depression, compared with 70.5% of men. Research evidence mirrors the finding that some DCWD returned to work more easily than others (Grobler 2018; Ramano et al. 2016; Tomlinson et al. 2009). A German study similarly found that female survivors of cancer had a higher rate of return to work than male survivors (85% vs. 73%) (Arndt et al. 2019). It is the first author’s experience in a clinical setting (at the insurer in this study) that more DCWD who are women engage in vocational rehabilitation and that women are more compliant with case management; therefore, a higher return to work for women is understandable.

The majority of Southern Africa’s workforce is within the prime working age of 25–54 years old (42.37%), while the minority is of the mature working age of 55–64 years old (6.8%) (Statistics South Africa, Risenga Maluleke 2021). The typical onset of depression is between 20 years and 30 years of age, with an average age of onset at 26 years in Southern Africa, which coincides with the beginning of the prime working life for individuals (Statistics South Africa, Risenga Maluleke 2021). Disability claimant with depression below 60 years old had a significantly higher chance of return to work, possibly because of them being within the prime working age. In addition, return to work allows DCWD to engage in the meaningful activity of work tasks and improves work capacity after a period of prolonged workplace absence because of their depression.

A higher disability benefit termination for DCWD was associated with a higher level of education. This association is supported by a recent Norwegian study, which concluded that higher educated workers had a higher likelihood of sustained return to work following long-term sick leave because of depression (Meling et al. 2023). While a 2000 South African study found similar associations between levels of education and successful return to work (Watt & Penn 2000), more recent African studies found no association (Masterson et al. 2023; Modise et al. 2024). A possible reason for the association may be that persons with a higher level of education find it easier to adjust to a work environment following a prolonged period off work.

A scoping review by De Vries et al. indicates that high job demands, together with low organisational support, resulted in longer duration of sick leave for persons with mental health disorders (De Vries et al. 2018). The authors further note that younger age (under 60) and support from co-workers and supervisors were predictors of successful and earlier return to work (De Vries et al. 2018). These predictors of successful return to work can also be noted in this study, as younger age (under 60 years) and high workplace support are indicated as positive prognostic factors for termination of disability benefit for return to work among DCWD.

Over recent years, vocational rehabilitation has shifted to have a more integrated approach involving an inter-disciplinary team and taking into consideration the sociodemographic, workplace and personal factors of DCWD (Saonatse, De Witt & Van Niekerk 2019). Case managers within the insurance industry aim to guide the process of return to work for DCWD to ensure a successful return to work with the assistance of an occupational therapist. DCWDs who have undergone rehabilitation have a disability benefit termination for return to work prevalence of 80%. A feasibility study carried out in the Netherlands noted that supporting disability claimants on long-term disability with vocational rehabilitation is a facilitator for return to work (De Geus et al. 2023). This result can be used in a practical setting to motivate further case management interventions during the initial 24 months of disability for DCWD. Return to work and reintegration within the workplace are complex and dynamic processes, and occupational therapists have the necessary skills and knowledge to navigate and guide this process while ensuring that the DCWD remains at the centre through using a client-centred approach.

The return-to-work process for DCWD is an interplay between the DCWD, the insurer’s disability case manager, the rehabilitation providers and the employer. Each role player has their own responsibility during this process, and each needs to consider the sociodemographic, workplace, vocational rehabilitation and illness-related factors when working towards the common goal of successful return to work for DCWD.

### Limitations of the study

As a period of this study included the first year of the coronavirus disease 2019 (COVID-19) pandemic (2020), the return to work of a percentage of claimants with depression who received a disability benefit may have been impacted. This was not specifically accounted for during data analysis. This study analysed data from only one insurer’s database in Southern Africa, which is a limited sample. Data from the insurer were limited to two genders only (men and women) and did not account for gender non-binary. As DCWD did not have the option of identifying as non-binary, they may have been misrepresented in the data. Factors analysed for association were limited to those included in the database and highlighted in previous studies (Ervasti et al. 2017); thus, there may have been other relevant associated factors that were not represented. The study utilised existing retrospective data from one insurer. While it is acknowledged that the insurer’s data capture process had been refined over several years and is subject to regulated audits, the authors did not verify the accuracy of the data received from the insurer.

### Implications for practice and future research

This study identified groups of DCWD who need to be assisted earlier in the disability claim process with interventions to ensure a more successful and permanent return to work. These interventions include engaging with employers (Human Resources as well as team leaders) earlier to ensure that support is provided to the DCWD during the rehabilitation process. Furthermore, there needs to be ongoing training sessions and engagements with the employer by the disability case manager and rehabilitation providers, such as occupational therapists and psychologists. Engagement can take the form of psychoeducation and upskilling for ongoing support and recommendations for reasonable accommodations for DCWD within the workplace.

The findings add evidence suggesting the insurance industry should adopt a more person-centred and holistic approach when assessing disability claims for DCWD, as the literature has identified multiple factors that are associated with a successful return to work across all domains. Taking these factors into account during the case management and rehabilitation processes, prolonged periods of workplace absence can be prevented. Lastly, the factors identified can assist in case management policy development within the insurance industry in South Africa and motivate further funding for vocational rehabilitation interventions and engagements with employers.

Further studies on the individual-related and specific illness-related factors for DCWD and the strength thereof can be undertaken to identify further factors for a successful return to work that may influence practice. Future qualitative research can also investigate reasons for a more successful return to work in certain groups of claimants. The methodology used in this study could be used in future studies for disability claimants with other common mental health disorders to determine the factors associated with successful return to work in these populations.

## Conclusion

This study identified DCWD who successfully returned to work during a 3-year period and gathered data on the strength of association of the sociodemographic, workplace and illness-related factors for return to work. It is concluded that gender (women), age (younger than 60 years old) and workplace support (higher levels of support) are all positive prognostic factors associated with return to work for DCWD.
